# Analysis of Outcomes in Adolescents and Young Adults With Pilonidal Disease

**DOI:** 10.3389/fsurg.2021.613605

**Published:** 2021-02-25

**Authors:** Mackenzie N. Abraham, Steven L. Raymond, Russell B. Hawkins, Atif Iqbal, Shawn D. Larson, Moiz M. Mustafa, Janice A. Taylor, Saleem Islam

**Affiliations:** ^1^Department of Surgery, University of Alabama at Birmingham School of Medicine, Birmingham, AL, United States; ^2^Department of Surgery, University of Florida College of Medicine, Gainesville, FL, United States; ^3^Department of Surgery, Baylor College of Medicine, Houston, TX, United States

**Keywords:** pilondal sinus, pilonidal disease, recurrence, wound complication, adolescents, young adults, surgery, management

## Abstract

**Purpose:** Numerous definitive surgical techniques exist for the treatment of pilonidal disease with varied recurrence rates and wound complications. Due to the wide array of techniques and lack of consensus on the best approach, we proposed to study our experience treating pilonidal disease in adolescents and young adults.

**Methods:** A retrospective analysis was conducted of patients 10–24 years old treated at a tertiary medical center from 2011 to 2016. Data including demographics, management, and outcomes were collected and analyzed. Primary outcome was recurrence of disease.

**Results:** One hundred and thirty three patients with pilonidal disease underwent operative management. Fifty one percent underwent primary closure and 49% healed by secondary intention with no significant difference in recurrence rates (primary 18%, secondary 11%; *p* = 0.3245). Secondary healing patients had significantly lower wound complication rates (primary 51%, secondary 23%; *p* = 0.0012). After accounting for sex, race, weight, and operative technique, age was predictive of disease recurrence with an adjusted odds ratio (OR) of 0.706 (0.560–0.888; *p* = 0.003). Age and sex were both predictive of wound complications. Older patients had decreased risk of wound complication (adjusted OR 0.806, 95% CI 0.684–0.951; *p* = 0.0105), and male patients had increased risk of wound complication (adjusted OR 2.902, 95% CI 1.001–8.409; *p* = 0.0497).

**Conclusion:** In summary, there is no significant difference in the recurrence rates between operative techniques for pilonidal disease. Older patients have decreased risk of recurrence following intervention. Wound complication rates are lower in patients undergoing secondary healing, though this may be better explained by differences in age and sex. Additional research investigating newer, minimally-invasive techniques needs to be pursued.

## Introduction

Pilonidal sinus disease is a chronic and recurring condition which occurs at the natal cleft. The name “pilonidal” comes from the Latin meaning “nest of hair” as these sinuses typically contain large amounts of hair (1). The etiology of the condition is not fully understood; however, factors such as loose hair in the region, pressure causing introduction of the hair into the skin, and the sensitivity of the skin at the natal cleft are thought to contribute to the disease (2, 3). For patients initially presenting with symptoms of acute infection with pilonidal sinus, incision and drainage is recommended and has shown to be curative in ~60% of patients (4–6). However, the remaining 40% of patients experience recurring infection, drainage, and pain which requires additional treatment.

The ideal treatment for chronic pilonidal sinus should have a low incidence of recurrence and complication while also minimizing pain and hospital stay (7). There are many different surgical treatments available: excision with primary midline closure, excision with flap or off-midline closure, and excision with secondary or open healing, including marsupialization. These methods vary on a wide range of factors such as recurrence, healing time, and infection rates (8–10). There is notably a trend toward less invasive procedures, such as video-assisted ablation, due to recurrence and complication associated with open operative procedures (11–14).

Due to the wide array of available treatments and the lack of consensus on the best approach, we conducted a retrospective analysis of pilonidal disease in adolescents and young adults at a large, academic medical center. Our goals were to better understand the outcomes of pilonidal disease from different approaches in management and identify which factors contribute to increased morbidity.

## Materials and Methods

### Study Design

This retrospective observational study was conducted at UF Health Shands Hospital, a 1,162-bed tertiary, academic medical center. The study was approved by the Institutional Review Board prior to initiation. Patients were identified using ICD-9-CM, ICD-10-CM, and CPT codes to ensure capture of all cases. The study staff then retrospectively reviewed the inpatient and outpatient electronic medical records of identified patients.

### Inclusion and Exclusion Criteria

Patients between 10 and 24 years of age who underwent management of pilonidal disease at the study institution from January 2011 to December 2016 were considered for inclusion in the study. Patients who underwent surgical intervention at an outside facility were excluded. Patients without at least one documented post-operative follow up visit were also excluded.

### Variables, Outcomes, and Definitions

Patient data including demographics, clinical characteristics, disease course, procedures, surgical management, and outcomes were collected from the electronic medical records of inpatient and outpatient encounters. Primary clinical outcome was recurrent pilonidal disease, defined as reformation of cyst or abscess anytime during follow up. Secondary clinical outcomes were length of hospital stay, number of post-operative visits, and wound complications including wound dehiscence, excess bleeding or drainage, and non-healing. Any wound requiring secondary intervention was included as a complication.

### Statistical Analysis

Data are presented as frequency and percentage for categorical variables and as mean and range for continuous variables. Analysis of variance and Student's *t*-tests were used for comparison of continuous parametric variables. Kruskal-Wallis and Mann-Whitney tests were used for comparison of continuous non-parametric variables. Fischer's exact test was used for comparison of categorical variables. Univariate and multivariate logistic regression models were created to predict recurrence and wound complications based on patient age, sex, operative technique, and other explanatory variables. For multivariate analyses, stepwise selection was utilized with a 0.1 significance threshold used for inclusion in the model. All significance tests were two-sided, with *p*-values <0.05 considered statistically significant. Statistical analyses were performed with Prism GraphPad Software (La Jolla, CA, USA) and SAS version 9.4 (Cary, NC, USA).

## Results

### Patient Demographics and Management

Two hundred forty-five patients were identified and met all study criteria. Overall cohort demographics are provided in Table 1. Overall mean age was 18.4 years (range 11–24). There was a slight predominance of females in the cohort (51%). Overall mean body mass index (BMI) was 29.4 (range 17.7–51.0).

**Table 1:** Patient demographics and clinical outcomes of operative and non-operative groups.

	**Total (*n* = 245)**	**Operative (*n* = 133)**	**Non-operative (*n* = 112)**	*p*-value^*^
Age in years, mean (range)	18.4 (11–24)	18.3 (11–24)	18.5 (13–24)	0.6198
Gender, *n* (%)				
Male	120 (49.0)	73 (54.9)	47 (42.0)	0.0543
Race, *n* (%)				
Black	38 (15.5)	15 (11.3)	23 (20.5)	0.0524
White	114 (46.5)	75 (56.4)	39 (34.8)	0.0008
Hispanic	13 (5.3)	6 (4.5)	7 (6.3)	0.5789
Other/unknown	80 (32.7)	37 (27.8)	43 (38.4)	0.1006
Weight in kg, mean (range)	84.7 (41–170)	82.5 (45.3–132.9)	87.3 (40.9–169.8)	0.0784
BMI, mean (range)	29.4 (17.7–51)	28.7 (17.7–46.1)	30.3 (19.7–51.0)	0.0841
Insurance status, *n* (%)				
Uninsured	29 (11.8)	8 (6.0)	21 (18.8)	0.0026
Medicaid	75 (30.6)	41 (30.8)	34 (30.4)	1.0000
Private	139 (56.7)	84 (63.2)	55 (49.1)	0.0287
Unknown	2 (0.8)	0 (0)	2 (1.8)	0.2080
Smoking, *n* (%)				
Yes	32 (13.1)	15 (11.3)	17 (15.2)	0.4473
No	196 (80.0)	110 (82.7)	86 (76.8)	0.2653
Unknown	17 (6.9)	8 (6.0)	9 (8.0)	0.6174
Symptoms, *n* (%)				
Pain	239 (97.6)	127 (95.5)	112 (100)	0.0328
Bleeding	57 (23.3)	42 (31.6)	15 (13.4)	0.0008
Persistent drainage	108 (44.1)	72 (54.1)	36 (32.1)	0.0008
Abscess formation	189 (77.1)	98 (73.7)	91 (81.3)	0.1723
Recurrent abscess	68 (27.8)	41 (30.8)	27 (24.1)	0.2554
Unknown	2 (0.8)	2 (1.5)	0 (0)	0.5016
Antibiotic use, *n* (%)	207 (84.5)	108 (81.2)	98 (87.5)	0.2205
I&D, n (%)				
Yes	157 (64.1)	65 (48.9)	92 (82.1)	<0.0001
Multiple I&D	48 (19.6)	26 (19.5)	22 (19.6)	1.0000
Occurred in ED	105 (42.9)	31 (23.3)	74 (66.1)	<0.0001
Definitive resection, *n* (%)	133 (54.3)	133 (100.0)	-	−
Primary closure	68 (27.8)	68 (51.1)	-	−
Off-midline	36 (14.7)	36 (27.1)	-	−
Midline	32 (13.1)	32 (24.1)	-	−
Secondary healing	65 (26.5)	65 (48.9)	-	−
Marsupialization	46 (18.8)	46 (34.6)	-	−
Vacuum assisted closure	3 (1.2)	3 (2.3)	-	−
Daily packing	16 (6.5)	16 (12.0)	-	−
Recurrence, *n* (%)	19 (7.8)	19 (14.3)	-	−
Multiple recurrence	8 (3.3)	8 (6.0)	-	−
Wound complication, *n* (%)	50 (20.4)	50 (37.6)	-	−
Hospital days, median (range)	1 (1–32)	1 (1–32)	-	−
Post-operative visits, median (range)	2 (1–29)	2 (1–29)	-	−

* *p-value describes relationship between the operative and non-operative groups*.

**Figure 1:**
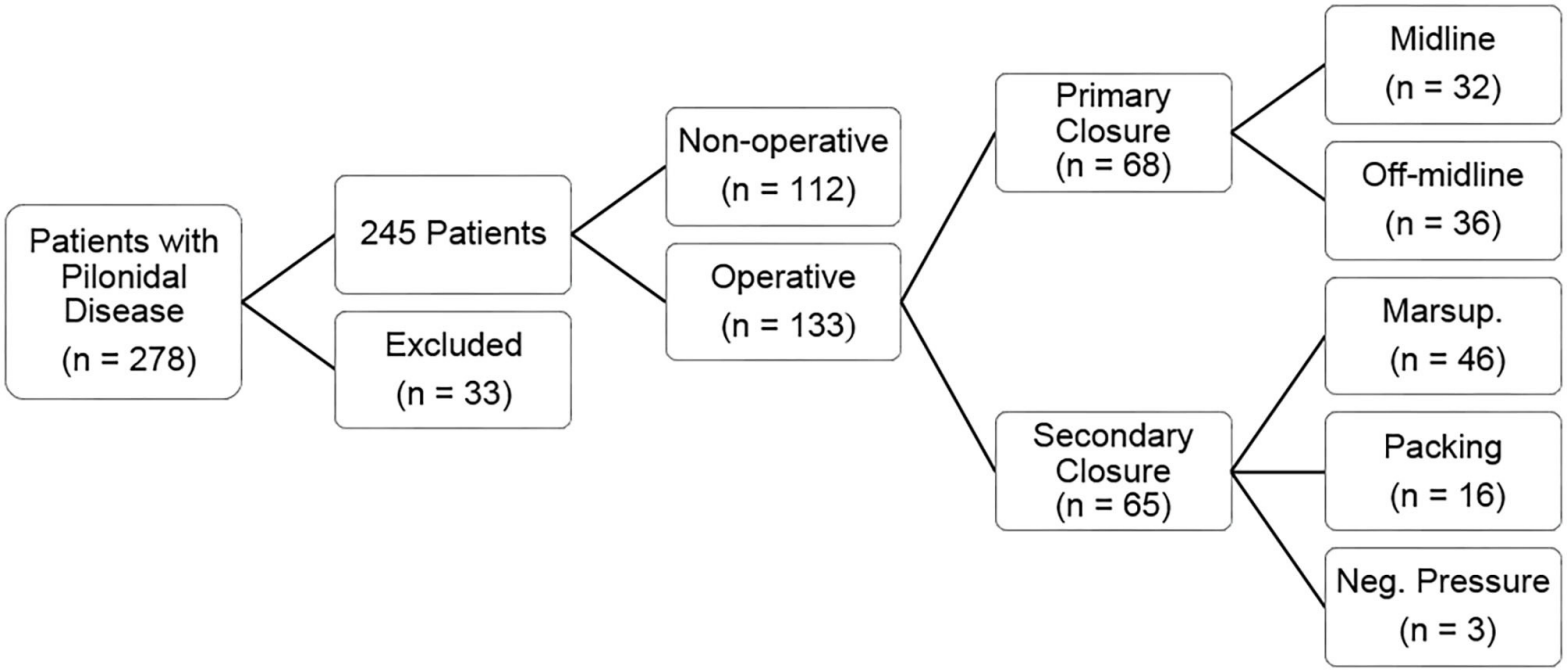
Flowchart displaying the breakdown of pilonidal disease management for patients in our cohort, including type of operative procedure.

One hundred twelve patients underwent non-operative management for pilonidal disease including 13 patients (12%) who had incision and drainage alone, 20 patients (18%) who received antibiotics, and 79 patients (71%) who had both incision and drainage and antibiotics. One hundred thirty-three patients underwent operative management for pilonidal disease including 65 patients (49%) who had incision and drainage of an abscess prior to surgical resection. The majority of patients that underwent operative intervention were diagnosed with pilonidal disease within a year of their operation. Among the operative group, 68 patients (51%) underwent excision with primary closure, whereas 65 patients (49%) were treated by marsupialization or cystectomy and allowed to heal by secondary intention (Figure 1). Primary closure was achieved using a midline approach for 32 patients and using off-midline approach for 36 patients. Off-midline approaches included Limberg, Karydakis, and Bascom techniques. Of those allowed to heal by secondary intention, 46 underwent marsupialization, 16 had packing placed, and three received negative pressure wound therapy devices.

Though there was no difference in gender or BMI between the treatment groups, we did find significant differences in age. Patients treated with secondary healing were noted to be older compared to those treated with primary healing (*p* < 0.0001, Table 2). Average age was significantly higher in the midline closure group as compared to the off-midline flap closure group (*p* = 0.0002, Table 3). Lastly, the marsupialization group was significantly older than all other cystectomy patients (*p* < 0.0001, Table 4).

**Table 2:** Patient demographics and clinical outcomes of primary and secondary closure groups.

	**Primary closure (*n* = 68)**	**Secondary closure (*n* = 65)**	***p*-value**
Age in years, mean (range)	16.8 (11–24)	19.8 (14–24)	<0.0001
BMI, mean (range)	29.9 (17.7–43.6)	28.5 (20.5–46.1)	0.7524
Recurrence, *n* (%)	12 (17.6)	7 (10.8)	0.3245
Wound complication, n (%)	35 (51.5)	15 (23.1)	0.0012
Hospital days, median (range)	1 (1–32)	1 (1–5)	0.0592
Post-operative visits, median (range)	3 (1–29)	2 (1–26)	0.1471

**Table 3:** Patient demographics and clinical outcomes of primary midline and off-midline closure groups.

	**Primary midline closure (*n* = 32)**	**Primary off-midline closure (*n* = 36)**	***p*-value**
Age in years, mean (range)	18.1 (13–24)	15.7 (11–22)	0.0002
BMI, mean (range)	27.6 (17.7–37.3)	30.1 (18.0–43.6)	0.2561
Recurrence, *n* (%)	4 (12.5)	8 (22.2)	0.3525
Wound complication, *n* (%)	13 (40.6)	22 (61.1)	0.1443
Hospital days, median (range)	1 (1–10)	1 (1–32)	0.3287
Post-operative visits, median (range)	2 (1–23)	5 (1–29)	0.0160

**Table 4:** Patient demographics and clinical outcomes of cystectomy and marsupialization groups.

	**Cystectomy (*n* = 87)**	**Marsupialization (*n* = 46)**	***p*-value**
Age in years, mean (range)	17.3 (11–24)	20.2 (15–24)	<0.0001
BMI, mean (range)	28.9 (17.7–43.6)	28.5 (20.5–46.1)	0.6047
Recurrence, *n* (%)	16 (18.4)	3 (6.5)	0.0723
Wound complication, *n* (%)	40 (46)	10 (21.7)	0.0081
Hospital days, median (range)	1 (1–32)	1 (1–1)	0.0097
Post-operative visits, median (range)	3 (1–29)	1 (1–14)	0.0001

### Clinical Outcomes

In total, 19 patients who underwent operative management for pilonidal disease experienced recurrence (14%). The average time to documented recurrence was 316.3 days. Of the 19 patients that had recurrence, 16 patients underwent at least one additional re-operation. The same surgical technique was performed in the majority of re-operations. There was no statistically significant difference in recurrence rates for patients who underwent primary closure compared to secondary healing (primary 18%, secondary 11%; *p* = 0.3245, Table 2). Surgical patients who underwent secondary healing had a significantly lower wound complication rate compared to those with excision and primary closure (primary 52%, secondary 23%; *p* = 0.0012, Table 2). Specifically, patients who underwent excision with off-midline primary closure had the highest percentage of wound complications (off-midline 61%, midline 41%, secondary 23%; *p* = 0.0007). Off-midline primary closure patients also had higher numbers of post-operative visits compared to the midline primary closure group (midline 2 visits, off-midline five visits; *p* = 0.0160, Table 3). Patients undergoing marsupialization had significantly reduced wound complication rates (*p* = 0.0081), shorter length of stay (*p* = 0.0097), and fewer post-operative visits (*p* = 0.0001) compared to all other surgical cystectomy patients (Table 4). When patients with <2 post-operative visits were excluded from the analysis, similar results were found. There remained no difference in recurrence rates when comparing operative techniques, and patient who underwent excision with secondary healing had a significant lower wound complication rate compared to those with excision and primary closure (primary 67%, secondary 39%; *p* = 0.0238). Additionally, those patients who underwent excision with off-midline primary closure still demonstrated the highest percentage of wound complications with 22 of 32 patients having complications.

Multivariate analyses of the variables age, sex, race, weight, and operative technique were performed to predict recurrence and wound complication. Age was the only variable to predict recurrence with older patients having decreased risk of recurrence (adjusted OR 0.706, 95% CI 0.560–0.888; *p* = 0.003). Age and sex were both predictive of wound complications. Older patients had decreased risk of wound complication (adjusted OR 0.806, 95% CI 0.684–0.951; *p* = 0.0105), and male patients had increased risk of wound complication (adjusted OR 2.902, 95% CI 1.001–8.409; *p* = 0.0497). Type of operative technique was not predictive of recurrence (adjusted OR 1.457, 95% CI 0.804–2.642; *p* = 0.2151) or wound complication (adjusted OR 0.984, 95% CI 0.631–1.535; *p* = 0.9477) in these multivariate models.

## Discussion

The results of this study indicate that while the overall recurrence rates of pilonidal disease are similar among primary and secondary closure techniques, wound complication rates were significantly higher among primary closure groups compared to secondary healing groups. Marsupialization was associated with decreased wound complications, shorter hospital length of stay, and fewer post-operative visits when compared to all other methods.

The study herein found no difference in recurrence between the secondary healing and primary closure groups (primary 18%, secondary 11%; *p* = 0.32; *n* = 133). Similarly, Fike et al. performed a single-institution retrospective analysis of patients aged 1–19 years old which demonstrated primary and secondary closure groups had no difference in recurrence rates (primary 25%, secondary 21%; *p* = 0.51; *n* = 120) (15). Our study did find that secondary healing was associated with reduced wound complications, most likely due to a reduction in risk of infection and wound dehiscence associated with primary closure.

When compared to all other methods, we found that marsupialization was associated with decreased wound complications, shorter hospital length of stay, and fewer post-operative visits. Previous studies show lower rates of recurrence and morbidity with marsupialization compared to other techniques, such as wide local excision and flap closure (16–19). Rouch et al. performed a multicenter retrospective analysis of adolescent patients undergoing marsupialization or wide local excision for pilonidal disease and demonstrated decreased recurrence among the marsupialization group (marsupialization 4%, wide local excision 31%; *p* = 0.03; *n* = 56) (16). The study herein showed marsupialization had a non-statistically significant decreased recurrence rate compared to cystectomy (marsupialization 7%, cystectomy 18%, *p* = 0.07; *n* = 133).

Numerous studies have concluded in favor of off-midline flap closure over midline closure, especially in cases of complex and recurring disease (3, 20–29). Ertan et al. randomized 100 patients with chronic pilonidal disease to primary midline closure or off-midline closure with a Limberg flap, and demonstrated shorter hospital stay (midline 4.6 days, off-midline 3.4 days; *p* = 0.005), time to complete healing (midline 15.7 days, off-midline 10.3 days; *p* = 0.001), mean time off work (midline 28.5 days, off-midline 15.8 days; *p* = 0.001), and wound infection rates (midline 20%, off-midline 6%; *p* = 0.03) for the off-midline flap technique (20). Off-midline or flap closure techniques are thought to address underlying causes of the condition and thus reduce recurrence by flattening the natal cleft and minimizing tension in the region (27, 30). Many of these previous studies are from single centers, as is ours, and focus on a single technique, reporting favorable short-term outcomes. Conversely, the study herein compared a variety of surgical methods and found that off-midline closure, including Limberg, Karydakis, and Bascom techniques, resulted in similar outcomes compared to midline closure. Differences in recurrence and wound complication rates between the different types of flap closure methods have been evaluated with varying results, though we did not examine this in our study due to small sample sizes in each group (24–26).

Trends toward less invasive options in management of pilonidal disease including laser hair epilation, phenol or fibrin injection, tract excision, and video-assisted ablation may point toward universal recognition of a failure of more invasive techniques in adequate disease management. At the time of the study period, video-assisted techniques for management of pilonidal disease were not being performed at the study institution, thus were not included in the analysis. In 2019, Milone et al. published that video-assisted ablation of pilonidal sinus has decreased recurrence rate compared to excision with secondary healing (video-assisted ablation 7.5%, standard sinusectomy 25%; *p* = 0.035; *n* = 107) (31). Milone et al. subsequently compared their experience with video-assisted ablation of pilonidal sinus vs. conventional Bascom cleft lift procedure and showed similar long-term recurrence rates (video-assisted ablation 24%, cleft lift 24%; *p* = 0.95; *n* = 141) and improved patient satisfaction (video-assisted ablation 8.9, cleft lift 7.8; *p* = 0.001; *n* = 141) (32). Esposito et al. recently reported on the use of pediatric endoscopic pilonidal sinus treatment and laser epilation for 59 patients with acute or chronic pilonidal sinus disease and showed a recurrence rate of 1.6% with maximum follow-up of 30 months (33).

In the multivariate analyses, age was predictive of recurrence and wound complication after accounting for sex, race, weight, and operative technique. Specifically, older females had the best outcomes. This observation may be partially explained by improved personal hygiene and compliance in older patients, as previous studies have shown that poor hygiene is associated with higher rates of recurrence and complication (15, 34). In addition, patients that require surgical intervention at a younger age may have more severe disease and prone to worse outcomes.

There are limitations in this study that bear discussion. This retrospective analysis is limited in scope by the information documented in the electronic medical record which excluded the ability to investigate the impact of laser hair removal techniques and other adjuvant therapies that may be available to patients at other institutions and impact outcomes of pilonidal disease. Furthermore, the study was unable to account for and measure patient satisfaction, time to complete healing, and other patient related factors which may impact the preferred treatment approach. Surgeon preference for the implemented operative technique may also play a role and introduce selection bias in the study. Patients were excluded from the study that had no documented post-operative follow up; however, the overall median number of post-operative visits was two visits, which may lead to an underestimation of recurrence and complication rates. The study design also prevented the ability to capture patients that may have followed up at other intuitions. It is important to note that the post-operative visits only capture visits with the surgeon after the initial operation and do not include visits with the patients' primary care providers and/or gastroenterologists within the same large healthcare system consisting of more than 700 outpatient providers. Based on this, the author has confidence in the reported outcomes, as a patient would be referred back to the surgical service if there were evidence of wound complications and/or recurrence. Additionally, a sub-analysis excluding patient with <2 post-operative visits demonstrated similar outcome results overall. Despite the inherent limitations, the large number of cases and multivariate analyses preformed help provide strength to growing body of literature involving the management of pilonidal disease.

In summary, there are multiple surgical techniques utilized for definitive management of pilonidal disease. Unfortunately, high rates of recurrence and wound complication persist when managing this complex condition. We found that female patients have decreased risk of wound complications following operative intervention for pilonidal disease, and older patients have decreased risk of recurrence and wound complications. Wound complication rates are lower in patients undergoing secondary healing compared to primary closure, though this observation is better explained by differences in age and sex.

## Data Availability Statement

The raw data supporting the conclusions of this article will be made available by the authors, without undue reservation.

## Ethics Statement

The studies involving human participants were reviewed and approved by University of Florida Institutional Review Board. Written informed consent from the participant or the participants' legal guardian/next of kin was not required to participate in this study in accordance with the national legislation and the institutional requirements.

## Author Contributions

AI, SL, MM, JT, and SI developed the hypothesis and designed the experiments. MA, SR, and RH collected the data and analyzed the results. MA, SR, and SI prepared the original manuscript. All authors reviewed and revised the final manuscript. No editorial support was used of the preparation of the manuscript.

## Conflict of Interest

The authors declare that the research was conducted in the absence of any commercial or financial relationships that could be construed as a potential conflict of interest.
